# Single Nucleotide Polymorphisms (SNP) and SNP-SNP Interactions of the Surfactant Protein Genes Are Associated With Idiopathic Pulmonary Fibrosis in a Mexican Study Group; Comparison With Hypersensitivity Pneumonitis

**DOI:** 10.3389/fimmu.2022.842745

**Published:** 2022-06-02

**Authors:** Ata Abbasi, Chixiang Chen, Chintan K. Gandhi, Rongling Wu, Annie Pardo, Moises Selman, Joanna Floros

**Affiliations:** ^1^ Cellular and Molecular Research Center, Cellular and Molecular Medicine Institute, Urmia University of Medical Sciences, Urmia, Iran; ^2^ Department of Pathology, Faculty of Medicine, Urmia University of Medical Sciences, Urmia, Iran; ^3^ Department of Public Health Science, Pennsylvania State University College of Medicine, Hershey, PA, United States; ^4^ Department of Pediatrics, Pennsylvania State University College of Medicine, Hershey, PA, United States; ^5^ Facultad de Ciencias, Universidad Nacional Autónoma de México, Mexico City, Mexico; ^6^ Unidad de Investigación, Instituto Nacional de Enfermedades Respiratorias “Ismael Cosio Villegas”, Mexico City, Mexico; ^7^ Department of Obstetrics & Gynecology, Pennsylvania State University College of Medicine, Hershey, PA, United States

**Keywords:** surfactant protein, single nucleotide polymorphism, idiopathic pulmonary fibrosis, hypersensitivity pneumonitis, SNP-SNP interactions

## Abstract

Surfactant proteins (SPs) are important for normal lung function and innate immunity of the lungs and their genes have been identified with significant genetic variability. Changes in quantity or quality of SPs due to genetic mutations or natural genetic variability may alter their functions and contribute to the host susceptibility for particular diseases. Alternatively, SP single nucleotide polymorphisms (SNPs) can serve as markers to identify disease risk or response to therapies, as shown for other genes in a number of other studies. In the current study, we evaluated associations of *SFTP* SNPs with idiopathic pulmonary fibrosis (IPF) by studying novel computational models where the epistatic effects (dominant, additive, recessive) of SNP-SNP interactions could be evaluated, and then compared the results with a previously published hypersensitivity pneumonitis (HP) study where the same novel models were used. Mexican Hispanic patients (IPF=84 & HP=75) and 194 healthy control individuals were evaluated. The goal was to identify SP SNPs and SNP-SNP interactions that associate with IPF as well as SNPs and interactions that may be unique to each of these interstitial diseases or common between them. We observed: 1) in terms of IPF, i) three single *SFTPA1* SNPs to associate with decreased IPF risk, ii) three *SFTPA1* haplotypes to associate with increased IPF risk, and iii) a number of three-SNP interactions to associate with IPF susceptibility. 2) Comparison of IPF and HP, i) three *SFTPA1* and one *SFTPB* SNP associated with decreased risk in IPF but increased risk in HP, and one *SFTPA1* SNP associated with decreased risk in both IPF and HP, ii) a number of three-SNP interactions with the same or different effect pattern associated with IPF and/or HP susceptibility, iii) one of the three-SNP interactions that involved SNPs of *SFTPA1*, *SFTPA2*, and *SFTPD*, with the same effect pattern, was associated with a disease-specific outcome, a decreased and increased risk in HP and IPF, respectively. This is the first study that compares the SP gene variants in these two phenotypically similar diseases. Our findings indicate that SNPs of all *SFTPs* may play an important role in the genetic susceptibility to IPF and HP. Importantly, IPF and HP share some SP genetic variants, suggesting common pathophysiological mechanisms and pathways regarding surfactant biogenesis, but also some differences, highlighting the diverse underlying pathogenic mechanisms between an inflammatory-driven fibrosis (HP) and an epithelial-driven fibrosis (IPF). Alternatively, the significant SNPs identified here, along with SNPs of other genes, could serve as markers to distinguish these two devastating diseases.

## Introduction

Idiopathic pulmonary fibrosis (IPF) is one of the most common interstitial diseases of unknown etiology and poor prognosis (1, 2). It is characterized by aberrant activation of the lung epithelium which provokes the increase and activation of the fibroblasts population that finally leads to the replacement of lung parenchyma with destructive fibrotic bundles leading to respiratory failure and death (3, 4). IPF is a chronic, progressive, irreversible, and usually lethal disease of middle-aged and elderly patients (5, 6). Multiple efforts on different aspects to find out the etiology of this disease have been made but with little progress (3, 7–10). The clinical course of IPF is heterogeneous and considerable overlap exists in presentation of IPF and chronic hypersensitivity pneumonitis (HP) (11).

Hypersensitivity pneumonitis (HP) is another type of interstitial lung disease caused by an exaggerated immune response to environmental antigens, such as fugal, bacterial or bird proteins (12). Although antigens that cause HP have been identified and are distributed worldwide, only a very small percent of the world population gets affected and the distribution among different nations is not similar (12). In Mexico, the pigeon breeder’s disease is the most common type of HP, caused by proteins from avian serum, feces, and feathers (12).

IPF and HP are different diseases with different etiologies. Validated risk factors for IPF include mainly aging and smoking; exposure to metal dust, wood dust, pesticide, and occupational history of farming or agriculture also increased the risk of IPF. Likewise, the risk of HP is associated mainly to the exposure to organic particles and varies with regional disparities in climate, occupational exposures, and environmental exposures, but not ethnicity. Respiratory viral infections, and high pesticide exposure, have been revealed as risk factors. Paradoxically, cigarette smoke reduces the risk of HP, but when smokers develop HP, they often follow a chronic fibro-proliferative course. However, there is a considerable overlap in pathophysiology and clinical presentation (6, 12–14). In fact, a previous study has shown that almost half of IPF patients, were, subsequently, diagnosed with chronic fibrotic HP, and often the clinical, functional and radiological behavior of both diseases are indistinguishable (13, 14). In both diseases, several members of the same family may be affected, indicating the role of genetic factors in the pathogenesis and progression of these diseases (15, 16). However, the interplay of genetics, environmental factors, and perhaps other factors is poorly understood.

Pulmonary surfactant and surfactant proteins (SPs), have been shown to play roles in host-defense functions, i.e. regulation of pro-inflammatory cytokine production, chemotaxis, and tissue repair (17–20), and in surfactant-related functions, i.e. lowering surface tension and stabilizing the alveoli. Hence, derangement in functional ability, structure, and/or levels of SPs (SP-A, SP-B, SP-C, SP-D) may contribute to the development of interstitial lung diseases, such as IPF and HP (21, 22). Alternatively, genetic polymorphisms of these important molecules may serve, along with other gene variants, as markers to distinguish these two diseases that share overlapping pathology and clinical presentation. Being able to distinguish these two diseases early on is of great importance. In this context, it has been recently reported a diagnostic algorithm which allow a better differential diagnosis between fibrotic HP and IPF (23). Accurate diagnosis is essential for appropriate treatment and management of these diseases. Currently, Nintedanib and pirfenidone, inhibitors of fibrosis pathways, are the treatments of choice for IPF (24). In contrast, HP treatment is based on corticosteroids and immunosuppressive therapy and importantly, administration of corticosteroids and immunosuppressants in some of the IPF patients can worsen their clinical condition, primarily those with severe telomere shortening, causing adverse effects and increased mortality (25). A better understanding of the pathobiology of fibrotic HP and of IPF as well as the genetic and molecular differences between them might help to identify biomarkers that allow distinguishing those patients who would benefit from antigen-exposure avoidance and immunosuppression (i.e., HP), from those with IPF in which the use of these drugs is not only unhelpful but increases the risk of hospitalization and death (12, 23, 25–27). There have been numerous examples in the literature where specific SNPs have been associated with disease susceptibility (28) or drug-response in certain disease populations (27, 29–31). For example, as reviewed and analyzed elsewhere (32) genotypes of molecules carrying certain SNPs were associated with response rates after imatinib treatment in patients with chronic myeloid leukemia. Furthermore, surfactant protein A2 genetic variants and genotypes of the donor lung have been associated with post-transplant clinical outcome i.e., survival in lung transplant patients (33).

The human SP-A is encoded by two similar genes, *SFTPA1* and *SFTPA2*, located on chromosome 10 (19, 34–36). Several genetic polymorphisms of each *SFTPA* gene, have been identified and characterized, and these are found with different frequencies in the general population (18, 19, 37–39). SP-A plays an important role in both innate lung host defense and surfactant-related processes (40) and the human variants may differentially affect these processes (19). SP-B, SP-C, and SP-D are each encoded by a single gene, *SFTPB, SFTPC*, and *SFTPD*, respectively (41), and several polymorphisms have been described for each of these genes (42–44). SP-D also plays a role in lung host defense and the primary role of SP-B and SP-C is in surfactant-related functions/processes. Furthermore, multiple studies showed associations of SPs genetic variants with various acute and chronic lung diseases including acute respiratory distress syndrome (ARDS) (43, 44), chronic obstructive pulmonary disease (COPD) (45, 46), cystic fibrosis (47), and other (44).

In a previous study, we showed associations of SP single nucleotide polymorphisms (SNPs) with IPF in a Mexican population (22). In addition, a number of studies identified significant associations between SP genetic variants or mutations and IPF, pointing to a potential role of SPs in the pathogenesis or specific processes of IPF (1, 7, 10, 48–52). Furthermore, to date many gene polymorphisms are known to be present in both IPF and HP interstitial lung disorders. Common genetic variants in TERC, DSP, MUC5B, ATP11A, FAM13A, and IVD described initially as risk gene factors for IPF also represent risk variants for HP, especially for the fibrotic HP (53, 54). Likewise, rare variants in telomere-related genes, such as TERT, TERC, DKC1, RTEL1, and PARN, have been identified in familial and sporadic IPF, and recently the same mutations were identified in patients with HP (55, 56).

In the present study, we used a statistical approach (57) where novel statistical models were studied to enable investigation of the epistatic effects of SNP-SNP interactions as has been done for other pulmonary diseases (21, 47, 58, 59). Using this novel approach, we reanalyzed IPF data that were previously published. The published study used regression analysis nearly 20 years ago (22). Next we compared these newly analyzed IPF data with our recently published HP data where the same novel statistical models were investigated (21). This comparison study is the first such study where SP variants were compared between IPF and HP. The objectives of the current study are: a) to re-analyze the existing IPF database (22), using the Wang’s newer analytical approach (57), to gain further insight into complex SNP-SNP interactions, and b) to compare the IPF and HP data of SNP-SNP interactions, from the current IPF study and our recently HP published work (21), to identify unique interactions to each disease. The rationale for the latter is that both IPF and HP are members of the ILD family and some unique interactions may help to differentiate these two diseases, while shared interactions may reveal some common pathways.

## Methods

### Study Population

The IPF and HP study population are the same as the ones described before (21, 22). Briefly, eighty-four and seventy-five unrelated patients were enrolled in the IPF and HP study groups, respectively. The diagnoses of both diseases were supported by clinical observation, pulmonary function, high-resolution computed tomography, and bronchoalveolar lavage findings (4, 12). In some cases, surgical biopsy was done to confirm the diagnosis. Patients with the diagnosis of other interstitial lung disease were excluded. All enrolled HP patients were classified to have a fibrotic HP (4). For both studies, one hundred and ninety-four healthy individuals served as controls. All study members were Hispanic Mexican individuals and enrolled at the National Institute of Respiratory Diseases in Mexico City. The protocol was approved by the Ethics Committee. The demographics and clinical characteristics of the study groups are shown in **Table 1**. Blood samples from enrolled subjects were collected and the genotyping was done using the PCR-RFLP method as described in the previous studies (21, 22).

**Table 1:** Demographics and clinical characteristics of the study groups.

Characteristic	Idiopathic pulmonary fibrosis (n = 84)	Hypersensitivity Pneumonitis (n = 75)	Healthy controls (n = 194)
Sex, male/female (%)	59/25 (70/30)	5/70 (8/92)	124/70(64/36)
Age (years)	62.3 ± 10.9	44 ± 13.2	41 ± 14.5
Nonsmokers/smokers (%)	54/30 (64/36)	61/14 (81/19)	103/91(53/47)
FVC % predicted	62.6 ± 14.6	56.6 ± 21.6	106.5 ± 11.3*
FEV1% predicted	65.6 ± 15.8	59.6 ± 21.7	99.7 ± 12.8*
FEV1/FVC%	88.0 ± 10.1	90.7 ± 8.5	79.3 ± 5.5*

*Performed in 122 healthy controls.

### Surfactant Protein Genes and Single Nucleotide Polymorphisms

We evaluated, as shown in **Table 2**, 17 SNPs of the SP genes using the single SNP model and the two and three-SNP-SNP interaction models described by Wang et al. (57). These included five from *SFTPA1*, four from *SFTPA2*, four from *SFTPB*, two from *SFTPC*, and two from *SFTPD.*

**Table 2:** SNPs of each surfactant protein gene analyzed in this study.

SFTPA1	SFTPA2	SFTPB	SFTPC	SFTPD
rs1059047	rs1059046	rs2077079	rs4715	rs721917
rs1136450	rs17886395	rs3024798	rs1124	rs2243639
rs1136451	rs1965707	rs1130866		
rs1059057	1965708	rs7316		
rs4253527				

### Statistical Analysis

As mentioned previously (57), we used a SNP–SNP interaction method in a case-control setting to study associations of SP genes SNPs with IPF. Wang et al. developed a computational model for detecting additive, dominant and epistatic effects by integrating quantitative genetic theory into a case-control design context (57). This approach integrates the principle of quantitative genetics, and decomposes the overall genetic effect of each SNP into different components: additive (a), dominant (d), and recessive (r) and can characterize high-order epistatic interactions. For example, consider two genes A and B, which may have four types of epistasis, additive x additive, additive x dominant, dominant x additive, dominant x dominant. These four types function differently to affect disease risk. For example, if a x a is important, this means that the two-marker genotype AABB (homozygote AA at gene A and homozygote BB at gene B) performs differently from the two-marker genotype AAbb (homozygote AA at gene A and homozygote bb at gene B). However, if d x d is significant, this means that double heterozygote AaBb performs differently from the other genotypes. Therefore, it is important to distinguish these four types of epistasis.

This SNP-SNP interaction approach (57) has been used and validated in studying associations of high order epistatic interactions with various acute and chronic pulmonary diseases (21, 47, 58, 59). The cases and controls were converted into a 2 × 2 contingency table and various types of epistatic interactions at different orders were tested. The p-value was adjusted for sex and smoking due to their modifying effects on both diseases. To account for multiple testing, false discovery rate (FDR) was set at 5%. The Cochran-Mantel-Haenszel test was used to calculate the Odds ratios (OR) with 95% of confidence interval (95%CI). All possible interactions for two- and three-SNP interaction models were tested and those with p value less than 0.05 are reported.

## Results

### IPF

#### Main Effect Analysis of the IPF Group (n = 84) vs the Control Group (n = 194)

In the present study, we observed significant differences in the studied groups with the single- and the three-SNP interaction model. We did not observe any significant SNP-SNP interactions in the two-SNP interaction model.

##### Single SNP Model

Three SNPs, rs1059047, rs1136450, and rs1059057 of *SFTPA1*, were each associated with decreased risk of IPF (OR: 0.42 to 0.46, p value: 0.008-0.010) (**Table 3**).

**Table 3:** IPF association with *SFTPA1* single SNPs.

	SNP	Odds ratio (OR)	OR range	adjusted-P value
1	rs1059047	0.461	0.28-0.75	0.010
2	rs1136450	0.422	0.25-0.7	0.008
3	rs1059057	0.442	0.27-0.7	0.008

##### Association of SNP-SNP Interaction with IPF in the Three-SNP Interaction Model

As previously described, each SNP can have additive, dominant or recessive effect on the disease phenotype and these are noted as a, d, and r, respectively. In our analysis, each SNP had dominant or additive effects, and no recessive effect was observed (**Supplementary Tables 1–6**).

#### Comparison of the IPF Group (n = 84) vs the Control Group (n = 194)

We found a total of 277 significant SNP-SNP interactions associated with IPF in the three-SNP model. The interactions are shown in detail in **Supplementary Tables 1–6**. Out of these 277 interactions, we observed the following. First, a) 30 interactions with two additive effects and one dominant effect. Four of these exhibited the axdxa effect pattern and 26 the dxaxa; b) 121 interactions with two dominant and one additive effect, (dxdxa (n=65), axdxd (n=27), dxaxd (n=29)) and c) 126 interactions with three dominant effects (dxdxd). Second, 44 were among SNPs of hydrophilic SPs (*SFTPA1, SFTPA2, SFTPD*) alone, 14 were among hydrophobic SPs (*SFTPB, SFTPC*) alone, and the others were among SNPs of both the hydrophilic and hydrophobic SPs. Third, most interactions (n=196) were associated with increased risk of IPF (p=0.001-0.046, OR= 1.48-11.4) and the remaining (n=81) were associated with decreased risk of IPF (p=0.001- 0.049, OR= 0.12-0.69). Among the interactions with increased risk of IPF, 7 were in the same SP gene (intragenic). Of these, 6 were in *SFTPA1* (4 with dxdxd, 1 with axdxd and 1 with dxdxa interactions effect) and 1 in *SFTPB* (with dxaxd interaction effect). Among the interactions with decreased risk of IPF, 2 were intragenic in the *SFTPB* with dxdxa interactions. No significant interactions were observed with recessive (r x r x r) or additive effects (a x a x a) only.

Overall, out of the 17 SNPs studied, 14 were found in SNP-SNP interactions shown to significantly associate with IPF in the three-SNP model. Of the 14 SNPs, six were SNPs of *SFTPA* (4 *SFTPA1* and 2 *SFTPA2*), 4 of *SFTPB*, 2 of *SFTPC* and 2 of *SFTPD.* Although all of these SNPs were frequently present in the significant SNP-SNP interactions, the rs1059046 of *SFTPA2* encoding amino acid 9 ((AA9), Asn>Thr) was the most frequently present followed by *SFTPC* SNPs. The frequency of each of the 14 SNPs found in significant SNP-SNP interactions associated IPF are shown in **Table 4**.

**Table 4:** Frequency of SNPs in significant SNP-SNP interactions.

SNP	Amino Acid (AA) No.	Surfactant Protein (SP)	frequency in interactions	Amino acid change
rs1136451	AA62	SP-A1	56	–
rs1136450	AA50	SP-A1	60	leu>val
rs1059047	AA19	SP-A1	56	val>Ala
rs1059057	AA133	SP-A1	55	–
rs1059046	AA9	SP-A2	76	Asn>Thr
rs1130866	AA131	SP-B	63	Thr131Ile
rs2077079	AA2	SP-B	60	His2Leu
rs3024798		SP-B	55	–
rs17886395	AA91	SP-A2	57	Pro>Aln
rs2243639	AA180	SP-D	52	Thr180Ala
rs4715	AA138	SP-C	71	Thr138Asn
rs1124	AA186	SP-C	74	Ser186Asn
rs7316		SP-B	43	–
rs721917	AA31	SP-D	53	Met31Thr

Amino acid (AA) designation for SP-A (36, 60) is based on the precursor molecule that includes the signal peptide. Amino designation for SP-B, SP-C, and SP-D does not include the signal peptide (https://www.ncbi.nlm.nih.gov/snp). After signal peptide cleavage both proSP-B and proSP-C undergo several peptide cleavages to give rise to the mature SP-B and SP-C, respectively.

Thirteen interactions had OR greater than 5 indicating a strong association with increased risk of IPF (**Table 5**). Among them one was an intragenic interaction of *SFTPB* SNPs (rs2077079, rs3024798, rs7316) and twelve interactions had two-SNPs with additive and one with dominant effect. In all but one, the *SFTPA1* SNP (rs1136450, (AA50), leu>val) was the one with the dominant effect.

**Table 5:** SNP interactions associated with IPF with odds ratio higher than 5.

Single nucleotide polymorphism (SNP)			interaction	Odds ratio (OR)	OR interval
rs2077079	rs3024798	rs7316	dxaxd	5.483	2.3-14.5
					
rs1136450	rs1136451	rs4715	dxaxa	11.430	2.5-107.1
rs1136450	rs1136451	rs1124		10.978	2.4-104.5
rs1136450	rs1059057	rs4715		5.964	2.1-19.1
rs1136450	rs1059057	rs1124		6.238	2.2-20
rs1136450	rs2077079	rs1130866		8.835	2.8-33.8
rs1136450	rs3024798	rs1130866		6.926	2.3-24.1
rs1136450	rs7316	rs4715		6.796	2.7-18.5
rs1136450	rs7316	rs1124		8.904	3.3-26.9
rs1136450	rs4715	rs1124		6.360	2.9-14.9
					
rs1059047	rs1136450	rs4715	axdxa	5.964	2.1-19.1
rs1059047	rs1136450	rs1124		6.027	2.1-19.3
rs4715	rs1124	rs2243639		9.266	2.3-55.1

#### Haplotype Analysis

**Figure 1:**
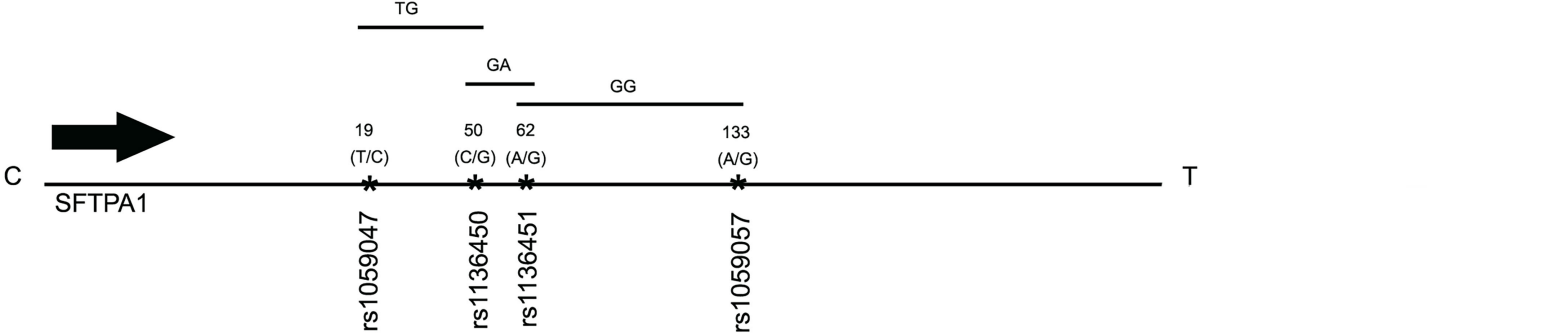
A schematic presentation of the *SFTPA1* gene SNPs is shown. The relative location of the gene is shown from centromere (C) to telomere (T). The numbers above the solid black line indicate the amino acid number with the corresponding nucleotide change shown in parenthesis. The SNP id is noted below the black line. The arrow indicates transcriptional orientation. The transmitted haplotypes (TG, GA, and GG) are shown in a two-SNP model and are associated with increased risk of IPF (OR: OR = 2.372, p = 0.004, OR = 2.368, p = 0.004 and OR = 2.265, p = 0.004, respectively). The TG haplotype is constituted by the rs1059047 Val (T) at codon 19 and the rs1136450 Val(G) at codon 50, the GA haplotype by the rs1136450 Val(G) at codon 50 and the rs1136451 Prol(A) at codon 62 and the GG by the rs1136451 Prol(G) at codon 62 and the rs1059057 Thr(G) at codon 133. Of note, the T/C and C/G alleles change the encoded amino acids at codons 19 Val/Ala and 50 Val/Leu, respectively, but the SNPs at codons 62 and 133 do not change the encoding amino acids (19).

Haplotype analysis showed that three haplotypes TG (rs1059047 x rs1136450), GA (rs1136450 x rs1136451) and GG (rs1136451 x rs1059057) of the *SFTPA1* were associated with increased risk of IPF (OR=2.372, p=0.004, OR=2.368, p=0.004 and OR=2.265, p=0.004, respectively) and all exhibited a dominant effect (**Table 6** and **Figure 1**). The dominant effect of, for example, the TG haplotype displays a higher risk of IPF compared to the combination of non-risk haplotypes (CC, TT, GG). This is also true for haplotypes GA and GG of *SFTPA1*.

**Table 6:** Haplotype association with IPF susceptibility.

	SNPi	SNPj	Risk haplotype	Odds ratio (OR)	OR range	Adjusted P value
1	rs1059047	rs1136450	TG	2.372	1.437-3.916	0.004
2	rs1136450	rs1136451	GA	2.368	1.434-3.910	0.004
3	rs1136451	rs1059057	GG	2.265	1.398-3.669	0.004

All SNPs shown are from SFTPA1.

### Comparison Between IPF (n = 84) and HP (n = 75) Using the Same Control Group (n = 194)

#### Comparison of SNP Associations in the Single-SNP Model

**Figure 2:**
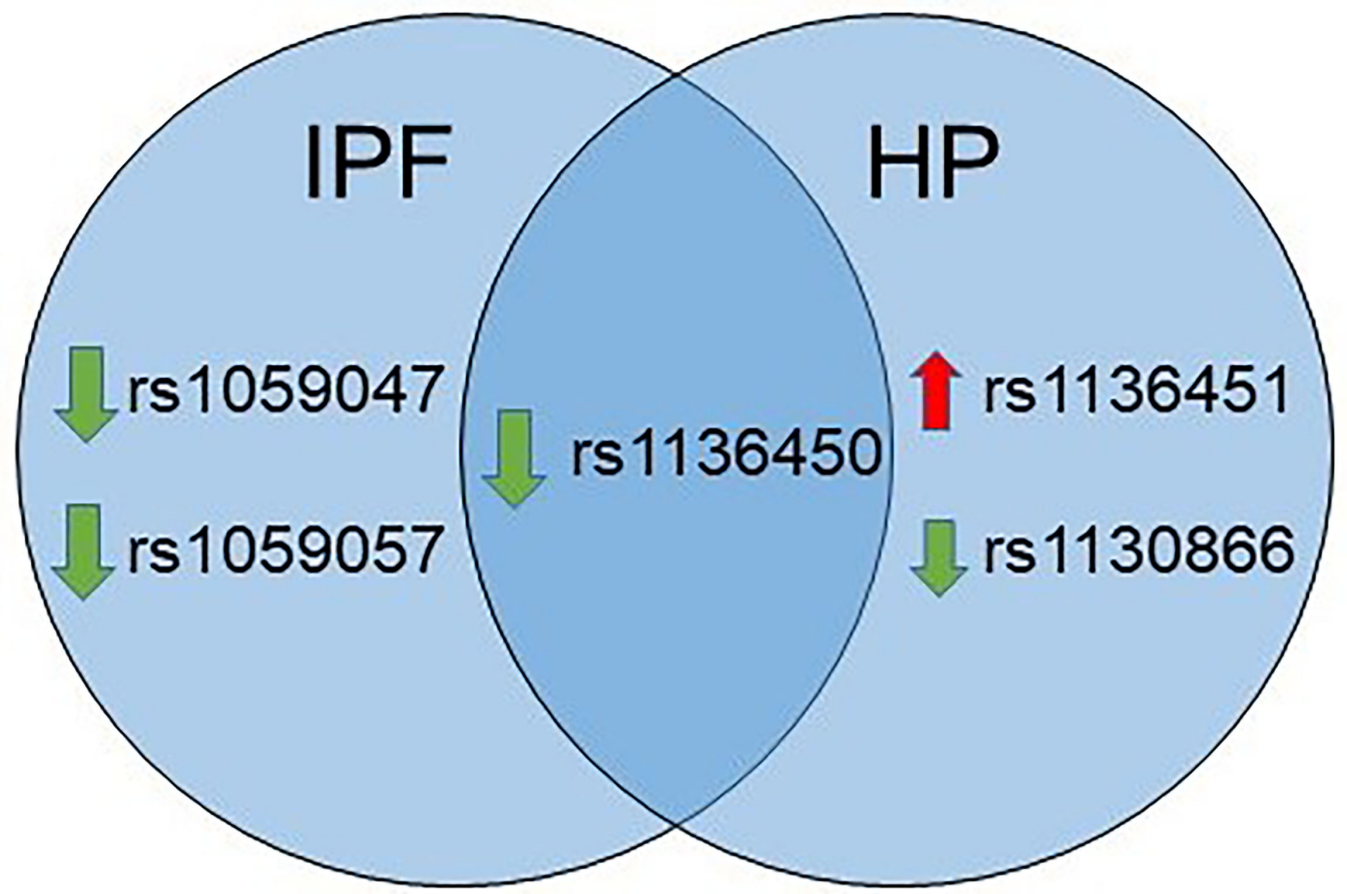
Venn diagram showing similarities and differences in association of SNPs with IPF and HP in a single SNP model. The green and red arrows besides SNPs show association of a given SNP with decreased and increased risk, respectively.

As shown in **Figure 2**, the rs1136450 of the *SFTPA1* was associated with a decreased risk for both diseases. The rs1059047 and the rs1059057 of the *SFTPA1* were associated with decreased risk of IPF, whereas the rs1136451 of the *SFTPA1* was associated with increased risk of HP. The rs1130866 of the *SFTPB* was associated with decreased risk of HP.

#### Common SNP-SNP Interactions Between IPF and HP After Comparison to the Same Control Group (n = 194) in the Three SNP Model

We studied the common SNP-SNP interactions in the three SNP model between the 277 interactions resulting from the comparison of the IPF group vs the control group (present study) and the 97 interactions resulting from the comparison of the HP group vs the control group in the three SNP model from our previous study (21). Of the 227 interactions in IPF comparison, as noted above, 81 interactions were associated with decreased risk of IPF, and the remaining 196 with increased risk of the disease. Of the 97 HP interactions, 68 and 29 were associated with decreased and increased risk of HP, respectively. However, in a large number of SNP-SNP interactions, the same SNPs were involved in IPF and HP. These SNP interactions exhibited either the same effect pattern between the two groups, IPF and HP, for example dxdxa (**Table 7**) or a different effect pattern between the two group comparisons i.e., dxdxa vs dxaxd (**Table 8**).

**Table 7:** Interactions with the same SNPs and the same pattern effect in IPF and HP patients.

Number	SNPs involved in interactions			pattern	OR for HP	OR for IPF
1	rs1059046	rs1136450	rs1136451	axdxd	0.08	0.33
	*SFTPA2*	*SFTPA1*	*SFTPA1*			
2	rs1059047	rs1136450	rs1136451	dxdxd	1.87	2.09
	*SFTPA1*	*SFTPA1*	*SFTPA1*			
3	rs1059047	rs2077079	rs3024798	dxdxd	2.070	2.36
	*SFTPA1*	*SFTPB*	*SFTPB*			
**4**	**rs1059046**	**rs1136450**	**rs721917**	**dxdxd**	**0.44**	**2.05**
	rs1059046	rs1136450	rs721917	dxdxa	3.66	2.37
	*SFTPA2*	*SFTPA1*	*SFTPD*			
5	rs1136450	rs1136451	rs721917	dxdxa	3.16	2.77
	*SFTPA1*	*SFTPA1*	*SFTPD*			
6	rs17886395	rs2077079	rs3024798	dxdxd	2.18	2
	*SFTPA2*	*SFTPB*	*SFTPB*			
7	rs1136450	rs1136451	rs721917	dxdxa	3.16	2.77
	*SFTPA1*	*SFTPA1*	*SFTPD*			
**8**	**rs1059046**	**rs2077079**	**rs4715**	**dxdxa**	**0.38**	**2.38**
	*SFTPA2*	*SFTPB*	*SFTPC*			
**9**	**rs3024798**	**rs4715**	**rs1124**	**dxdxd**	**0.45**	**2.54**
	*SFTPB*	*SFTPC*	*SFTPC*			
**10**	**rs1130866**	**rs4715**	**rs1124**	**dxdxa**	**0.33**	**2.7**
	*SFTPB*	*SFTPC*	*SFTPC*			
11	rs1136450	rs2077079	rs1130866	dxdxd	2.14	2.11
	*SFTPA1*	*SFTPB*	*SFTPB*			
12	rs1136450	rs3024798	rs1130866	dxdxd	1.89	2.09
	*SFTPA1*	*SFTPB*	*SFTPB*			
13	rs1136451	rs2077079	rs1124	dxdxd	1.87	2.1
	*SFTPA1*	*SFTPB*	*SFTPC*			
14	rs17886395	rs1136451	rs2077079	dxdxa	3.75	2.86
	*SFTPA2*	*SFTPA1*	*SFTPB*			
15	rs2077079	rs3024798	rs721917	dxdxa	7.86	3.7
	*SFTPB*	*SFTPB*	*SFTPD*			
16	rs3024798	rs721917	rs2243639	axdxd	3.43	2.57
	*SFTPB*	*SFTPD*	*SFTPD*			
17	rs1059046	rs17886395	rs1124	dxdxa	0.38	0.46
	*SFTPA2*	*SFTPA2*	*SFTPC*			
18	rs1059046	rs1136451	rs4715	dxdxd	0.36	0.53
	*SFTPA2*	*SFTPA1*	*SFTPC*			
19	rs17886395	rs3024798	rs1130866	dxdxa	0.33	0.306
	*SFTPA2*	*SFTPB*	*SFTPB*			
20	rs17886395	rs4715	rs721917	dxdxa	0.29	0.35
	*SFTPA2*	*SFTPC*	*SFTPD*			
21	rs1059047	rs3024798	rs1130866	dxdxa	0.24	0.43
	*SFTPA1*	*SFTPB*	*SFTPB*			
22	rs1136450	rs2077079	rs3024798	axdxd	0.2	0.29
	*SFTPA1*	*SFTPB*	*SFTPB*			
23	rs1136450	rs4715	rs1124	axdxd	0.12	0.32
	*SFTPA1*	*SFTPC*	*SFTPC*			
24	rs1136450	rs3024798	rs721917	dxdxd	0.44	0.69
	*SFTPA1*	*SFTPB*	*SFTPD*			
25	rs1136450	rs1136451	rs4715	dxdxd	0.36	0.63
	*SFTPA1*	*SFTPA1*	*SFTPC*			
26	rs1130866	rs4715	rs1124	axdxd	0.33	0.32
	*SFTPB*	*SFTPC*	*SFTPC*			
27	rs1136451	rs3024798	rs1130866	dxdxa	0.29	0.48
	*SFTPA1*	*SFTPB*	*SFTPB*			
28	rs2077079	rs3024798	rs1130866	dxdxa	0.24	0.19
	*SFTPB*	*SFTPB*	*SFTPB*			

Interactions with different disease associations are in bold; OR, odds ratio.

**Table 8:** Interactions with the same SNPs and different effect pattern in IPF and HP patients.

No	Interactions			HP		IPF	
				pattern	OR	pattern	OR
1	rs1059046	rs1130866	rs721917	dxdxd	0.473	axdxa	0.117
	*SFTPA2*	*SFTPB*	*SFTPD*				
2	rs1059046	rs1130866	rs2243639	dxdxd	0.348	dxaxa	0.202
	*SFTPA2*	*SFTPB*	*SFTPD*				
3	rs1059046	rs17886395	rs1136450	dxaxd	0.27	axdxd	0.285
	rs1059046	rs17886395	rs1136450	dxdxa	0.274		
	*SFTPA2*	*SFTPA2*	*SFTPA1*				
4	rs1059046	rs17886395	rs1130866	dxdxa	0.27	axdxd	0.332
	*SFTPA2*	*SFTPA2*	*SFTPB*				
5	rs1059046	rs1136451	rs1130866	dxdxa	0.18	axdxd	0.368
	*SFTPA2*	*SFTPA1*	*SFTPB*				
6	rs1059046	rs3024798	rs4715	dxdxa	0.4	axdxd	0.389
	*SFTPA2*	*SFTPB*	*SFTPC*				
7	rs1059046	rs1124	rs721917	dxdxd	0.409	dxaxd	0.208
	*SFTPA2*	*SFTPC*	*SFTPD*				
8	rs1059046	rs3024798	rs721917	dxdxd	0.56	axdxd	0.327
	*SFTPA2*	*SFTPB*	*SFTPD*				
9	rs1136450	rs3024798	rs4715	dxdxa	0.34	axdxd	0.389
	*SFTPA1*	*SFTPB*	*SFTPC*				
10	rs1059047	rs1130866	rs2243639	axdxd	3.2	dxdxd	1.51
	*SFTPA1*	*SFTPB*	*SFTPD*				
11	rs1059046	rs1059047	rs2077079	axaxd	6.5	dxaxa	4.221
						dxdxa	2.515
						dxdxd	1.503
	*SFTPA2*	*SFTPA2*	*SFTPB*				
12	rs1059046	rs1059047	rs2243639	dxaxd	3.51	dxdxd	1.787
	*SFTPA2*	*SFTPA1*	*SFTPD*				
13	rs1136451	rs1130866	rs2243639	dxdxa	3.11	dxdxd	1.556
	*SFTPA1*	*SFTPB*	*SFTPD*				
14	rs17886395	rs1059047	rs1136451	dxdxa	5.96	dxdxd	1.995
	*SFTPA2*	*SFTPA1*	*SFTPA1*				
15	rs17886395	rs2077079	rs1130866	dxdxd	2.048	dxdxa	0.298
	*SFTPA2*	*SFTPB*	*SFTPB*				
16	rs1136450	rs2077079	rs1130866	dxdxd	2.14	axdxd	0.373
	*SFTPA1*	*SFTPB*	*SFTPB*				
17	rs1136450	rs3024798	rs2243639	dxdxd	1.96	axdxd	0.38
	*SFTPA1*	*SFTPB*	*SFTPD*				
18	rs1059047	rs1136450	rs721917	axaxa	0.053	dxdxd	1.506
	*SFTPA1*	*SFTPA1*	*SFTPD*				
19	rs1059046	rs2077079	rs3024798	axdxd	0.2	dxdxa	3.018
						dxaxd	3.589
						dxdxd	1.458
	*SFTPA2*	*SFTPB*	*SFTPB*				
20	rs1059046	rs17886395	rs2077079	axaxd	0.15	dxdxa	2.983
						dxdxd	1.807
	*SFTPA2*	*SFTPA2*	*SFTPB*				
21	rs1059046	rs17886395	rs1136450	dxdxa	0.274	dxdxd	1.756
	rs1059046	rs17886395	rs1136450	dxaxd	0.277		
	*SFTPA2*	*SFTPA2*	*SFTPA1*				
22	rs1059046	rs17886395	rs3024798	axaxd	0.073	dxdxd	1.565
	*SFTPA2*	*SFTPA2*	*SFTPB*				
23	rs1059046	rs17886395	rs1124	dxaxd	0.228	dxdxd	1.731
	rs1059046	rs17886395	rs1124	dxdxa	0.387		
	*SFTPA2*	*SFTPA2*	*SFTPC*				
24	rs1059046	rs17886395	rs1130866	dxdxa	0.270	dxdxd	1.502
	*SFTPA2*	*SFTPA2*	*SFTPB*				
25	rs1059046	rs3024798	rs4715	dxdxa	0.407	dxdxd	2.293
	*SFTPA2*	*SFTPB*	*SFTPC*				
26	rs1136450	rs1136451	rs4715	dxdxd	0.369	dxaxa	11.43
	*SFTPA1*	*SFTPA1*	*SFTPC*				
27	rs1136450	rs1136451	rs1124	dxdxd	0.502	dxaxa	10.98
	*SFTPA1*	*SFTPA1*	*SFTPC*				
28	rs1136450	rs2077079	rs3024798	axdxd	0.2	dxdxd	2.592
	*SFTPA1*	*SFTPB*	*SFTPB*				
29	rs1136450	rs4715	rs1124	axdxd	0.124	dxaxa	6.36
						dxdxa	3.448
						dxdxd	1.615
	*SFTPA1*	*SFTPC*	*SFTPC*				
30	rs1136451	rs3024798	rs1130866	dxdxa	0.292	dxdxd	1.858
	*SFTPA1*	*SFTPB*	*SFTPB*				
31	rs17886395	rs1136451	rs1130866	dxdxa	0.303	dxdxd	2.941
	*SFTPA2*	*SFTPA1*	*SFTPB*				
32	rs17886395	rs3024798	rs1130866	dxdxa	0.332	dxdxd	1.778
	*SFTPA2*	*SFTPB*	*SFTPB*				
33	rs17886395	rs1059047	rs1130866	dxdxa	0.294	dxdxd	1.673
	*SFTPA2*	*SFTPA1*	*SFTPB*				
34	rs2077079	rs3024798	rs2243639	axaxd	0.216	dxdxa	3.457
	*SFTPB*	*SFTPB*	*SFTPD*				
35	rs3024798	rs1130866	rs2243639	dxdxd	0.50	dxdxa	2.171
	*SFTPB*	*SFTPB*	*SFTPD*				
36	rs1059046	rs1136450	rs1136451	dxaxd	0.07	dxdxd	1.915
	rs1059046	rs1136450	rs1136451	dxdxa	3.551		
	*SFTPA2*	*SFTPA1*	*SFTPA1*				
37	rs1136451	rs2077079	rs3024798	dxaxd	0.162	dxdxd	1.661
	rs1136451	rs2077079	rs3024798	axdxd	9.198		
	*SFTPA1*	*SFTPB*	*SFTPB*				

OR, odds ratio.

##### Same SNPs and Same Pattern Effect in IPF and HP

We observed 28 interactions that involved the same SNPs (n=28) that exhibited the same effect pattern (n=29); Interaction #4 (**Table 7**) exhibited two different effect patterns. Of the 28 interactions, 13 of them were associated with decreased risk in both diseases and 12 of them with increased risk in both diseases. However, 4 interactions shown in bold in **Table 7**, involving the same SNPs and exhibiting the same pattern effect were associated with a decreased risk in HP and an increased risk in IPF indicating disease specificity. Moreover, one of these four interactions (rs1059046, rs1136450, rs721917 of *SFTPA2, SFTPA1, SFTPD*, respectively), noted as #4 in **Table 7**, exhibited two different patterns (dxdxd, dxdxa) in both IPF and HP, with the dxdxd (shown in bold) pattern being associated with an outcome that seemed to be disease-specific i.e., with a decreased risk in HP and an increased risk in IPF.

##### Same SNPs and Different Pattern Effect in IPF and HP

The interactions with the same SNPs (n=37) but with different pattern effects exhibited altered susceptibility in the two diseases and are shown in **Table 8**. For example, interactions #26 and #27, rs1136450 x rs1136451 x rs4715 and rs1136450 x rs1136451 x rs1124 (**Table 8**) with the dxaxa pattern were strongly associated with IPF (OR=11.43, 10.98, respectively), but the same SNP interactions in the dxdxd pattern were both associated with low risk of HP (OR=0.36 and 0.5, respectively). The rs1136450 x rs4715 x rs1124 (interaction #29, **Table 8**) is another interaction which was associated with increased risk of IPF in the dxaxa, dxdxa and dxdxd patterns (OR=6.36, 3.44 and 1.61, respectively) but associated with decreased risk of IPF and HP in the axdxd pattern (interaction #23, **Table 7**).

Of note, rs1059057 and rs7316 were not present in any of the common SNP-SNP interactions.

## Discussion

Surfactant proteins (SPs) are important in normal lung function and also in innate immunity of the lungs. Changes in quantity or quality of SPs due to genetic alterations may alter their functions, whether in host defense and/or surfactant-related processes, and contribute to the host susceptibility for particular diseases. SP-A, for example, plays an important role in innate host defense against various pathogens, irritants, and other (40) and its natural genetic variability may differentially affect these processes (19). In the current study, we evaluated associations of *SFTP* SNPs with IPF using a novel statistical method and compared the results of the current study with a previously published HP study (21). The goal here was to find SNPs and SNP-SNP interactions that are unique to each of these interstitial lung diseases or common between them. Importantly, IPF is an epithelial-driven fibrosis and is typically progressive, while HP is an immune inflammatory-driven disease, often evolving to fibrosis, and may also develop a progressive fibrotic phenotype. We observed, 1) in terms of IPF: i) three single *SFTPA1* SNPs to associate with decreased IPF risk, ii) three *SFTPA1* haplotypes to associate with increased IPF risk, and iii) a number of three-SNP interactions to associate with IPF susceptibility. 2) After comparison of IPF and HP, i) three *SFTPA1* and one *SFTPB* SNP were found to associate with decreased or increased risk in IPF and HP and one *SFTPA1* SNP to associate with decreased risk in both IPF and HP, ii) a number of three-SNP interactions with the same or different effect pattern associated with IPF and/or HP susceptibility; iii) one of the three-SNP interactions that involved SNPs of *SFTPA2*, *SFTPA1*, and *SFTPD*, with the same effect pattern, was associated with a disease-specific outcome, a decreased and increased risk in HP and IPF, respectively. The findings of the current study may provide an example to start differentiating these two diseases based on their genetic background.

For the current study, we took advantage of the same ethnic background of cases for both diseases (IPF and HP) and controls, i.e. All study subjects were Mexican Hispanic patients and all of them were enrolled from the same center. We hoped that this will help identify genetic patterns that in turn may enable distinguishing these two diseases or at the very least provide a paradigm for further work where genetic models could be used to find disease-specific markers.

### IPF

The *SFTPA1* gene is shown in the present study, *via* different analyses, including the use of a single SNP model, haplotype analysis and SNP-SNP interactions, to associate with IPF susceptibility. One of its SNPs, rs1136450, was the most frequently observed *SFTPA1* SNP in the 3-SNP interactions. This SNP was found to be significant either by itself, as part of significant haplotypes, or part of significant 3-SNP interactions and has been found previously to associate with susceptibility with other pulmonary diseases including community acquired pneumonia (61), cystic fibrosis (47), acute respiratory distress of the newborn (59) and pediatric acute and chronic respiratory failure (58, 62). The rs1136450 SNP encodes (leu/val) amino acid 50 (AA50), and it is located within the collagen-like region of SP-A1. In the absence of the collagen-like region, functional defects of surfactant are observed as well as the absence of an extracellular structural form of surfactant, the tubular myelin (63). This region is also important for the oligomerization and stability of SP-A (64). This SNP change is SP-A1 variant-specific and not gene-specific. All of the most frequently found and studied SP-A1 variants (6A, 6A^3^, and 6A^4^) have a leucine at AA50 except the 6A^2^ variant that has a valine. On the other hand, all frequently found and studied SP-A2 variants have a valine (19, 38, 39). These two amino acids (Leu/Val), although they share a lot of similarities, as they are both essential non-polar amino acids with aliphatic side chains and neutral charge at pH 7.4, they may however provide differential sites for proteases. Proteases are often highly specific in their proteolytic activity, and even a conservative substitution as this one, provided by the rs1136450 SNP, may have a significant differential effect on the proteolylic process, as demonstrated in detailed studies for the matrix metalloproteinases family (65). Whether and how the AA50 change imparted by this SNP differentially affects any of the studied properties affected by the collagen-like region or provides preferential sites for various enzymes remains to be determined.

Of interest, a previous analysis of the same cohort using the traditional logistic regression analysis showed association of three SNPs of the *SFTPA1*, rs4253527 (AA219_T), rs1136450 (AA50_C), and rs1136451 (AA62_G) with increased risk of IPF (22). In the present study, the “G” allele of the rs1136450 SNP was associated with decreased risk of IPF. Although these findings appear to contradict one another, both studies do identify the same significant SNPs. Also, this comparison provides an opportunity for a cautionary note where one needs to take into consideration the method of assigning, reference and alternate alleles of a given SNP. Wang et al.’s approach employed in the present study uses the dbSNP database of the NCBI (66) to assign the reference and the alternate alleles, whereas, in the logistic regression analysis, alleles were assigned based on the frequencies in the studied group, i.e., the more frequent allele in that study group was the reference allele. Furthermore, in the current study, we did not observe association of the rs4253527 (AA219_T) with IPF either in the single-SNP or in the three-SNP model. This may be due to the difference in statistical approaches used for the two studies. Although we did not observe association of rs1136451 in the single-SNP model either, this SNP was observed in several SNP-SNP interactions (**Table 1**).

However, what is of considerable interest are the observations made regarding associations with relative risk or protection of IPF. In the single SNP model, the *SFTPA1* SNP (rs1136450) and two other *SFTPA1* SNPs (rs1059047, rs1059057) were shown to associate with decreased risk but haplotypes that included the rs1136450 and/or the other two SNPs were associated with increased risk. Moreover, a number of 3-SNP interactions, where the rs1136450 had a dominant effect and any other two SNPs had an additive effect, exhibited a high odds ratio indicating risk. However, when the rs1136450 in 3-SNP interactions was found with different effect patterns, the interactions were associated with either increased or decreased risk. An example to demonstrate this is depicted in the 3-SNP interactions, #9 and #28 in **Table 8**, where in the former the pattern effect is axdxd and in the latter is dxdxd, where the rs1136450 has additive (a) and dominant (d) effect, respectively. These different effect patterns of the rs113650, were associated with decreased (axdxd) and increased (dxdxd) risk of IPF, respectively. A similar effect pattern observation in 3-SNP interactions was made for the *SFTPA2* rs1059046 (interactions, 3 & 21, 4 & 24, 6 & 25, **Table 8**). We postulate that the effect pattern, whether dominant (d) or additive (a) (no recessive effect was observed in this study), plays an important role in disease susceptibility. Although what contributes to a SNP to exhibit a different effect pattern is not entirely clear, we speculate that the overall cellular microenvironment, its interaction with other SNPs, and other unknown factors contribute to the specific effect pattern and its consequences, as it may be assessed by the strength of the given association with disease susceptibility. For example, different effect patterns among the same SNPs were observed, where each was associated with varying degree of IPF susceptibility as assessed by the odds ratio. Interaction #11 (**Table 8**) exemplifies this point. Three different effect patterns observed and each was associated with increased IPF risk albeit each exhibited a different odds ratio (range 1.5-4.2). These observations collectively indicate that fully understanding SNP-SNP interactions that may alter the risk of an individual to a disease, is a challenging problem (67, 68) due to the phenomenon of epistasis where the combined effect of one or more genes/SNPs on the phenotype could not have been predicted by their separate effects (69). Thus, this type of observations requires further consideration.

The central pathogenesis path of IPF is a progressive deposition of fibrotic tissue in the lungs secondary to dysregulated activity of the alveolar epithelium to repeated injury (7). Actually, mutations in genes encoding surfactant proteins, have been identified in adults with the phenotype of pulmonary fibrosis through a gain-of-toxic function mechanism (70). In the present study, we show that the *SFTP* genes play a role in IPF as shown by the numerous 3-SNP significant interactions that included SNPs of all the *SFTP* genes. Although, single SNPs or haplotypes of *SFTPA1* exhibited significant association with IPF susceptibility, no SNPs of other *SFTP* genes showed any significant associations. Considering the vital role of SP-A in innate immunity and host responses of the lung to foreign particles, these findings are not surprising. Of interest, *SFTPA1*, compared to *SFTPA2*, has been shown to be more efficient in surfactant lipid reorganization and in preventing surfactant inhibition by serum proteins, indicating that the importance of this gene in IPF may in part be due to its dual role in host defense and surfactant-related activities. Because the 3-SNP interactions involved SNPs from all *SFTPs*, we postulate that collectively *SFTP* variants contribute to IPF in ways that we are not able, to yet fully understand. Further research is needed in studying the impact of the actual SNP-SNP interactions on levels and biophysical/biochemical properties of SPs in appropriate biological models. This may shed light into the complexities of their interactions and advance our understanding of these interactions on complex diseases, such as IPF.

### Comparison of IPF and HP

When we compared findings of the current IPF study with a previously published HP study (21), using the same statistical approach, and cohort as control, a number of similarities and differences were identified between these interstitial lung diseases. Although one single *SFTPA1* SNP (rs1136450), was found to associate with decreased risk in both diseases, other *SFTPA1* SNPs were associated with IPF or HP disease-specific susceptibility. Thus, these point to a potential use of these SNPs as markers to distinguish between these two diseases. The *SFTPA1* significant SNPs that change the encoded amino acid may affect functional aspects of the protein variant. The significance of the rs1136450 has been discussed above under IPF. The rs1059047 of *SFTPA1* that associates with decreased risk in IPF, changes amino acid 19 (Val/Ala). This amino acid may or may not be part of all the mature SP-A1 molecules (60) and therefore it is unclear whether and how this may affect the processing of the precursor protein and/or the functional capability of the mature molecules that contain this amino acid.

Moreover, one *SFTPB* SNP (rs1130866) was associated specifically with decreased risk in HP. The SP-B protein plays a crucial role in surfactant function and some mutations are not compatible with life (71). The *SFTPB* SNP noted above is responsible for a missense codon (ACT/ATT) that changes amino acid 131 from a Threonine (ACT) to Isoleucine (ATT) and eliminates an N-linked glycosylation site (Asp^129^-Gln-Thr^131^) (72). Although amino acid 131 is part of the N-terminal peptide of the SP-B preprotein and not the mature SP-B, animal models have shown that the alleles of this SNP differentially affect the number of lamellar bodies, an extracellular structural form of surfactant, surface tension and levels of SP-B (73). Whether this SNP modifies any surfactant properties in humans and whether the consequences of these contribute to HP susceptibility is unknown. Its location is near a mutation hotspot and whether this, under certain circumstances, impacts other events is currently unknown (74).

Furthermore, we observed four 3-SNP interactions to exhibit a disease specific outcome. These interactions involved the same SNPs with similar effect patterns and associated with increased and decreased risk of IPF and HP, respectively (**Table 7**). These interactions involved SNPs from all *SFTPs*, with two of these (interactions #9 and 10) containing SNPs only from the hydrophobic surfactant proteins (*SFTPB and SFTPC*), one (interaction #4) contained SNPs only from the hydrophilic proteins (*SFTPA2, SFTPA1, SFTPD*) and one (interaction #8) contained SNPs from both groups (*SFTPA2, SFTPB, SFTPC*). Collectively, these indicate a role of all the surfactant proteins in the susceptibility of these diseases. Furthermore, the disease-specific lung microenvironment may alter the susceptibility of the host (75) and thus SNP interactions of the same SNPs with the same effect pattern may lead to different outcomes, as shown with the 3-SNP interactions in the present study. These interactions indicate that is plausible to distinguish the two similar interstitial lung diseases (IPF and HP), based on genetic interactions. Furthermore, we observed a number of 3-SNP interactions, with the same SNPs but with different effect patterns, to associate with IPF and HP disease susceptibility (**Table 8**). These observations are difficult to understand at the current time, as there may be a nonlinear relationship (dominant) between the gene products and disease outcomes, due perhaps to the gene dosage/imbalance and other sources of “more than-additive genetic interactions” that may lead to variable phenotypes of either over-, under- or non-function of the gene products in a given disease state (76).

The strengths of the current study are: a) the use of a newer statistical analysis to study SNP-SNP interactions with adjustment of important variables such as age, sex, and smoking status, b) enrollment of a patient population and control study groups with a similar ethnic background from the same center; therefore, the population structure of the studied groups may not be a major issue. Although, the second strength maybe a limitation (i.e., whether it can be generalized to include other groups of a different race or ethnicity), to our current knowledge ethnicity is not a significant risk factor as both diseases occur similarly in different ethnic groups as reported worldwide. However, there is limited information to determine whether differences in the frequency of the surfactant protein genetic variants, epigenetics, or differences in the SNP-SNP effect patterns studied here exist among different groups that could potentially point to different underlying processes in the pathogenesis of these disease. Thus, it is prudent to replicate the present findings in a larger sample size by case control studies with clinically confirmed cases of IPF and HP of other groups of heterogeneous non-Hispanic patients from various ethnicities in order to validate and strengthen the differential diagnostic potential of the identified SNPs and SNP interactions.

In conclusion, SNPs of all *SFTPs* appear to play an important role in identifying disease susceptibility in IPF and HP interstitial lung diseases. Similarities between these two diseases with regards to the surfactant protein genes variants were observed in haplotypes or in 3-SNP interactions, as well as differences highlighting the different underlying pathogenic mechanisms between an inflammatory-driven fibrosis (HP) and an epithelial-driven fibrosis (IPF). In addition, the disease-specific associations of the SP polymorphisms hold the potential for these SNPs to be used as markers to distinguish between these two diseases. The information obtained in the current study was enabled *via* the use of newer/novel statistical methods to study models of single SNPs as well as SNP-SNP interactions where epistasis could be addressed. *SFTPA1* and *SFTPB* SNPs might be of particular interest for future studies, where these could be used as markers either individually or together with other biomarkers in an attempt to distinguish between these two similar diseases. This is of great importance as it could help downstream decision-making of diagnosis and disease-specific therapies of these two devastating diseases.

## Data Availability Statement

The datasets presented in this study can be found in online repositories. The names of the repository/repositories and accession number(s) can be found below:

dbSNP, accession number: 1063345.

## Ethics Statement

The studies involving human participants were reviewed and approved by National Institute of Respiratory Diseases in Mexico City. The patients/participants provided their written informed consent to participate in this study.

## Author Contributions

AA analyzed the data and wrote the original draft of the manuscript. CC performed the statistical analysis. CG contributed to the manuscript writing. MS and AP were responsible for sample collection and patient screening as noted in previous publications and contributed in manuscript writing. RW worked closely with CC and guided the statistical analysis. JF supervised the entire project and worked closely with all co-authors and especially with AA to ensure quality of data analysis and finalize the manuscript. All authors contributed to the article and approved the submitted version.

## Funding

This work was supported by NIH HL34788 to JF.

## Conflict of Interest

The authors declare that the research was conducted in the absence of any commercial or financial relationships that could be construed as a potential conflict of interest.

## Publisher’s Note

All claims expressed in this article are solely those of the authors and do not necessarily represent those of their affiliated organizations, or those of the publisher, the editors and the reviewers. Any product that may be evaluated in this article, or claim that may be made by its manufacturer, is not guaranteed or endorsed by the publisher.

## Abbreviations

IPF, Interstitial pulmonary fibrosis; HP, Hypersensitivity Pneumonitis; SP, surfactant protein; *SFTPA1*, gene encoding *SP-A1*; *SFTPA2*, gene encodi ng *SP-A2*; *SFTPB*, gene encoding SP-B; *SFTPC*, gene encoding SP-C; *SFTPD*, gene encoding SP-C; SNPs, single nucleotide polymorphisms.
