# Supplementary material for: Single Nucleotide Polymorphisms (SNP) and SNP-SNP Interactions of the Surfactant Protein Genes Are Associated With Idiopathic Pulmonary Fibrosis in a Mexican Study Group; Comparison With Hypersensitivity Pneumonitis
Source: Front Immunol. 2022 Jun 2;13:842745. doi: 10.3389/fimmu.2022.842745 (PMC9201215; doi:10.3389/fimmu.2022.842745)
Supplement: Supplementary file 1 [file Table_1.docx]

Table 1: showing significant results from three SNP-SNP interaction analysis with axdxa pattern

|  | SNPi | SNPj | SNPk | unadjust-P | odds_ratio | lower_limit | upper_limit | adjusted-P(fdr) |
| --- | --- | --- | --- | --- | --- | --- | --- | --- |
| 1 | rs1059046 | rs1130866 | rs721917 | 0.000 | 0.117 | 0.035 | 0.339 | 0.002 |
| 2 | rs1059047 | rs1136450 | rs4715 | 0.000 | 5.964 | 2.140 | 19.122 | 0.015 |
| 3 | rs1059047 | rs1136450 | rs1124 | 0.000 | 6.027 | 2.151 | 19.361 | 0.015 |
| 4 | rs4715 | rs1124 | rs2243639 | 0.000 | 9.266 | 2.330 | 55.149 | 0.019 |

Table 2: showing significant results from three SNP interaction analysis with dxaxa pattern

|  | SNPi | SNPj | SNPk | unadjust-P | odds_ratio | lower_limit | upper_limit | adjusted-P(fdr) |
| --- | --- | --- | --- | --- | --- | --- | --- | --- |
| 1 | rs1059046 | rs1059047 | rs2077079 | 0.003 | 4.221 | 1.550 | 12.645 | 0.039 |
| 2 | rs1059046 | rs1059057 | rs2077079 | 0.001 | 4.344 | 1.598 | 12.987 | 0.029 |
| 3 | rs1059046 | rs1059057 | rs3024798 | 0.004 | 4.186 | 1.480 | 13.131 | 0.049 |
| 4 | rs1059046 | rs1130866 | rs2243639 | 0.001 | 0.202 | 0.062 | 0.593 | 0.029 |
| 5 | rs1059046 | rs7316 | rs1124 | 0.003 | 2.962 | 1.361 | 6.741 | 0.049 |
| 6 | rs17886395 | rs721917 | rs2243639 | 0.001 | 4.322 | 1.713 | 11.817 | 0.015 |
| 7 | rs1059047 | rs1136451 | rs1130866 | 0.002 | 0.152 | 0.031 | 0.565 | 0.029 |
| 8 | rs1059047 | rs7316 | rs4715 | 0.002 | 3.250 | 1.494 | 7.398 | 0.029 |
| 9 | rs1059047 | rs4715 | rs1124 | 0.000 | 3.344 | 1.659 | 7.006 | 0.009 |
| 10 | rs1136450 | rs1136451 | rs4715 | 0.000 | 11.430 | 2.532 | 107.182 | 0.005 |
| 11 | rs1136450 | rs1136451 | rs1124 | 0.000 | 10.978 | 2.382 | 104.509 | 0.008 |
| 12 | rs1136450 | rs1059057 | rs4715 | 0.000 | 5.964 | 2.140 | 19.122 | 0.005 |
| 13 | rs1136450 | rs1059057 | rs1124 | 0.000 | 6.238 | 2.226 | 20.058 | 0.005 |
| 14 | rs1136450 | rs2077079 | rs1130866 | 0.000 | 8.835 | 2.764 | 33.826 | 0.002 |
| 15 | rs1136450 | rs3024798 | rs1130866 | 0.000 | 6.926 | 2.307 | 24.096 | 0.005 |
| 16 | rs1136450 | rs7316 | rs4715 | 0.000 | 6.796 | 2.757 | 18.502 | 0.000 |
| 17 | rs1136450 | rs7316 | rs1124 | 0.000 | 8.904 | 3.327 | 26.973 | 0.000 |
| 18 | rs1136450 | rs4715 | rs1124 | 0.000 | 6.360 | 2.885 | 14.939 | 0.000 |
| 19 | rs1136451 | rs7316 | rs4715 | 0.000 | 3.965 | 1.798 | 9.222 | 0.008 |
| 20 | rs1136451 | rs7316 | rs1124 | 0.000 | 4.582 | 1.982 | 11.402 | 0.005 |
| 21 | rs1136451 | rs4715 | rs1124 | 0.002 | 2.912 | 1.450 | 6.048 | 0.029 |
| 22 | rs1136451 | rs721917 | rs2243639 | 0.002 | 3.885 | 1.553 | 10.233 | 0.029 |
| 23 | rs1059057 | rs7316 | rs4715 | 0.002 | 3.250 | 1.494 | 7.398 | 0.029 |
| 24 | rs1059057 | rs4715 | rs1124 | 0.000 | 3.344 | 1.659 | 7.006 | 0.009 |
| 25 | rs1130866 | rs4715 | rs2243639 | 0.000 | 0.178 | 0.065 | 0.452 | 0.004 |
| 26 | rs1130866 | rs1124 | rs2243639 | 0.001 | 0.232 | 0.085 | 0.588 | 0.015 |

Table 3: showing significant results from three SNP interaction analysis with dxdxa pattern

|  | SNPi | SNPj | SNPk | unadjust-P | odds_ratio | lower_limit | upper_limit | adjusted-P(fdr) |
| --- | --- | --- | --- | --- | --- | --- | --- | --- |
| 1 | rs1059046 | rs17886395 | rs2077079 | 0.000 | 2.983 | 1.678 | 5.418 | 0.002 |
| 2 | rs1059046 | rs17886395 | rs7316 | 0.000 | 0.330 | 0.187 | 0.567 | 0.001 |
| 3 | rs1059046 | rs17886395 | rs1124 | 0.004 | 0.460 | 0.261 | 0.797 | 0.027 |
| 4 | rs1059046 | rs1059047 | rs2077079 | 0.001 | 2.515 | 1.422 | 4.526 | 0.011 |
| 5 | rs1059046 | rs1059047 | rs7316 | 0.000 | 0.322 | 0.182 | 0.556 | 0.001 |
| 6 | rs1059046 | rs1136450 | rs721917 | 0.004 | 2.377 | 1.271 | 4.548 | 0.029 |
| 7 | rs1059046 | rs1136451 | rs1124 | 0.002 | 0.444 | 0.251 | 0.771 | 0.020 |
| 8 | rs1059046 | rs1059057 | rs2077079 | 0.000 | 2.724 | 1.528 | 4.954 | 0.006 |
| 9 | rs1059046 | rs1059057 | rs7316 | 0.000 | 0.334 | 0.188 | 0.577 | 0.001 |
| 10 | rs1059046 | rs2077079 | rs3024798 | 0.001 | 3.018 | 1.538 | 6.070 | 0.008 |
| 11 | rs1059046 | rs2077079 | rs4715 | 0.001 | 2.384 | 1.422 | 4.039 | 0.008 |
| 12 | rs1059046 | rs2077079 | rs1124 | 0.000 | 2.444 | 1.446 | 4.180 | 0.008 |
| 13 | rs1059046 | rs2077079 | rs721917 | 0.000 | 4.823 | 2.462 | 9.853 | 0.000 |
| 14 | rs1059046 | rs2077079 | rs2243639 | 0.000 | 3.179 | 1.730 | 5.993 | 0.002 |
| 15 | rs1059046 | rs1130866 | rs4715 | 0.001 | 2.353 | 1.412 | 3.958 | 0.008 |
| 16 | rs1059046 | rs1130866 | rs1124 | 0.001 | 2.256 | 1.355 | 3.783 | 0.011 |
| 17 | rs1059046 | rs7316 | rs721917 | 0.001 | 0.329 | 0.156 | 0.667 | 0.011 |
| 18 | rs1059046 | rs7316 | rs2243639 | 0.002 | 0.379 | 0.192 | 0.731 | 0.020 |
| 19 | rs17886395 | rs1059047 | rs4715 | 0.000 | 0.271 | 0.113 | 0.602 | 0.007 |
| 20 | rs17886395 | rs1059047 | rs1124 | 0.000 | 0.240 | 0.102 | 0.525 | 0.002 |
| 21 | rs17886395 | rs1136450 | rs2077079 | 0.006 | 2.130 | 1.207 | 3.826 | 0.035 |
| 22 | rs17886395 | rs1136451 | rs2077079 | 0.000 | 2.868 | 1.623 | 5.172 | 0.003 |
| 23 | rs17886395 | rs1059057 | rs4715 | 0.000 | 0.271 | 0.113 | 0.602 | 0.007 |
| 24 | rs17886395 | rs1059057 | rs1124 | 0.000 | 0.208 | 0.088 | 0.457 | 0.001 |
| 25 | rs17886395 | rs2077079 | rs1130866 | 0.000 | 0.298 | 0.159 | 0.545 | 0.001 |
| 26 | rs17886395 | rs3024798 | rs1130866 | 0.000 | 0.306 | 0.162 | 0.562 | 0.001 |
| 27 | rs17886395 | rs1130866 | rs721917 | 0.002 | 2.516 | 1.335 | 4.845 | 0.020 |
| 28 | rs17886395 | rs4715 | rs1124 | 0.005 | 2.375 | 1.245 | 4.643 | 0.034 |
| 29 | rs17886395 | rs4715 | rs721917 | 0.002 | 0.356 | 0.176 | 0.701 | 0.018 |
| 30 | rs17886395 | rs1124 | rs721917 | 0.006 | 0.421 | 0.213 | 0.816 | 0.036 |
| 31 | rs1059047 | rs1136450 | rs1059057 | 0.004 | 2.871 | 1.361 | 6.423 | 0.027 |
| 32 | rs1059047 | rs1136451 | rs2077079 | 0.000 | 2.922 | 1.654 | 5.263 | 0.002 |
| 33 | rs1059047 | rs1136451 | rs3024798 | 0.005 | 2.198 | 1.232 | 3.950 | 0.034 |
| 34 | rs1059047 | rs1059057 | rs2077079 | 0.001 | 2.371 | 1.367 | 4.172 | 0.013 |
| 35 | rs1059047 | rs1059057 | rs1130866 | 0.002 | 0.381 | 0.195 | 0.719 | 0.017 |
| 36 | rs1059047 | rs1059057 | rs4715 | 0.001 | 0.249 | 0.096 | 0.590 | 0.008 |
| 37 | rs1059047 | rs1059057 | rs1124 | 0.001 | 0.250 | 0.097 | 0.592 | 0.008 |
| 38 | rs1059047 | rs1059057 | rs721917 | 0.005 | 2.707 | 1.313 | 5.786 | 0.034 |
| 39 | rs1059047 | rs3024798 | rs1130866 | 0.005 | 0.436 | 0.232 | 0.799 | 0.034 |
| 40 | rs1059047 | rs4715 | rs1124 | 0.005 | 2.432 | 1.267 | 4.785 | 0.032 |
| 41 | rs1136450 | rs1136451 | rs721917 | 0.001 | 2.779 | 1.411 | 5.646 | 0.016 |
| 42 | rs1136450 | rs2077079 | rs7316 | 0.003 | 0.505 | 0.312 | 0.812 | 0.026 |
| 43 | rs1136450 | rs2077079 | rs2243639 | 0.000 | 3.078 | 1.720 | 5.639 | 0.002 |
| 44 | rs1136450 | rs7316 | rs1124 | 0.002 | 2.358 | 1.334 | 4.239 | 0.020 |
| 45 | rs1136450 | rs4715 | rs1124 | 0.000 | 3.448 | 1.752 | 7.038 | 0.003 |
| 46 | rs1136451 | rs1059057 | rs2077079 | 0.000 | 2.595 | 1.479 | 4.627 | 0.007 |
| 47 | rs1136451 | rs3024798 | rs1130866 | 0.008 | 0.480 | 0.269 | 0.846 | 0.043 |
| 48 | rs1136451 | rs1130866 | rs721917 | 0.002 | 2.658 | 1.394 | 5.197 | 0.018 |
| 49 | rs1136451 | rs7316 | rs721917 | 0.003 | 0.389 | 0.195 | 0.761 | 0.025 |
| 50 | rs1136451 | rs4715 | rs1124 | 0.006 | 2.305 | 1.225 | 4.434 | 0.035 |
| 51 | rs1059057 | rs3024798 | rs1130866 | 0.005 | 0.436 | 0.232 | 0.799 | 0.034 |
| 52 | rs1059057 | rs4715 | rs1124 | 0.005 | 2.432 | 1.267 | 4.785 | 0.032 |
| 53 | rs2077079 | rs3024798 | rs1130866 | 0.000 | 0.194 | 0.098 | 0.368 | 0.000 |
| 54 | rs2077079 | rs3024798 | rs721917 | 0.000 | 3.700 | 2.038 | 6.884 | 0.000 |
| 55 | rs2077079 | rs3024798 | rs2243639 | 0.000 | 3.457 | 1.940 | 6.335 | 0.000 |
| 56 | rs2077079 | rs721917 | rs2243639 | 0.008 | 0.441 | 0.228 | 0.839 | 0.043 |
| 57 | rs3024798 | rs1130866 | rs7316 | 0.001 | 0.465 | 0.285 | 0.751 | 0.011 |
| 58 | rs3024798 | rs1130866 | rs2243639 | 0.004 | 2.171 | 1.246 | 3.825 | 0.028 |
| 59 | rs1130866 | rs4715 | rs1124 | 0.002 | 2.708 | 1.395 | 5.421 | 0.017 |
| 60 | rs1130866 | rs4715 | rs2243639 | 0.003 | 0.397 | 0.200 | 0.762 | 0.026 |
| 61 | rs1130866 | rs1124 | rs2243639 | 0.007 | 0.437 | 0.229 | 0.811 | 0.039 |
| 62 | rs1130866 | rs721917 | rs2243639 | 0.001 | 0.328 | 0.165 | 0.639 | 0.008 |
| 63 | rs7316 | rs1124 | rs2243639 | 0.003 | 0.392 | 0.200 | 0.748 | 0.026 |
| 64 | rs4715 | rs1124 | rs721917 | 0.001 | 2.600 | 1.390 | 4.965 | 0.015 |
| 65 | rs4715 | rs1124 | rs2243639 | 0.006 | 2.358 | 1.244 | 4.607 | 0.035 |

Table 4: showing significant results from three SNP interaction analysis with axdxd pattern

|  | SNPi | SNPj | SNPk | unadjust-P | odds_ratio | lower_limit | upper_limit | adjusted-P(fdr) |
| --- | --- | --- | --- | --- | --- | --- | --- | --- |
| 1 | rs1059046 | rs17886395 | rs1136450 | 0.000 | 0.285 | 0.140 | 0.566 | 0.007 |
| 2 | rs1059046 | rs17886395 | rs1130866 | 0.001 | 0.332 | 0.163 | 0.653 | 0.017 |
| 3 | rs1059046 | rs17886395 | rs721917 | 0.003 | 2.447 | 1.297 | 4.651 | 0.047 |
| 4 | rs1059046 | rs1059047 | rs1130866 | 0.000 | 0.291 | 0.136 | 0.593 | 0.007 |
| 5 | rs1059046 | rs1136450 | rs1136451 | 0.001 | 0.337 | 0.163 | 0.676 | 0.027 |
| 6 | rs1059046 | rs1136451 | rs1130866 | 0.002 | 0.368 | 0.182 | 0.721 | 0.031 |
| 7 | rs1059046 | rs1059057 | rs1130866 | 0.000 | 0.239 | 0.108 | 0.502 | 0.003 |
| 8 | rs1059046 | rs3024798 | rs4715 | 0.001 | 0.389 | 0.212 | 0.705 | 0.025 |
| 9 | rs1059046 | rs3024798 | rs721917 | 0.000 | 0.327 | 0.161 | 0.633 | 0.013 |
| 10 | rs17886395 | rs2077079 | rs7316 | 0.003 | 0.412 | 0.217 | 0.767 | 0.047 |
| 11 | rs1059047 | rs1136450 | rs1059057 | 0.002 | 2.976 | 1.407 | 6.677 | 0.037 |
| 12 | rs1136450 | rs1059057 | rs1130866 | 0.001 | 0.318 | 0.145 | 0.663 | 0.026 |
| 13 | rs1136450 | rs2077079 | rs3024798 | 0.000 | 0.295 | 0.144 | 0.586 | 0.007 |
| 14 | rs1136450 | rs2077079 | rs1130866 | 0.003 | 0.373 | 0.189 | 0.723 | 0.039 |
| 15 | rs1136450 | rs2077079 | rs2243639 | 0.000 | 0.299 | 0.140 | 0.612 | 0.010 |
| 16 | rs1136450 | rs3024798 | rs4715 | 0.002 | 0.389 | 0.204 | 0.729 | 0.031 |
| 17 | rs1136450 | rs3024798 | rs721917 | 0.001 | 0.334 | 0.157 | 0.679 | 0.023 |
| 18 | rs1136450 | rs3024798 | rs2243639 | 0.003 | 0.380 | 0.188 | 0.747 | 0.042 |
| 19 | rs1136450 | rs4715 | rs1124 | 0.001 | 0.328 | 0.156 | 0.656 | 0.021 |
| 20 | rs2077079 | rs1130866 | rs4715 | 0.003 | 2.363 | 1.321 | 4.290 | 0.039 |
| 21 | rs2077079 | rs721917 | rs2243639 | 0.000 | 2.603 | 1.554 | 4.413 | 0.006 |
| 22 | rs3024798 | rs1130866 | rs4715 | 0.000 | 3.447 | 1.876 | 6.457 | 0.003 |
| 23 | rs3024798 | rs1130866 | rs1124 | 0.000 | 3.284 | 1.792 | 6.121 | 0.003 |
| 24 | rs3024798 | rs721917 | rs2243639 | 0.000 | 2.572 | 1.515 | 4.406 | 0.007 |
| 25 | rs1130866 | rs4715 | rs1124 | 0.000 | 0.323 | 0.172 | 0.589 | 0.004 |
| 26 | rs7316 | rs4715 | rs2243639 | 0.000 | 3.021 | 1.760 | 5.289 | 0.003 |
| 27 | rs7316 | rs1124 | rs2243639 | 0.000 | 2.976 | 1.739 | 5.194 | 0.003 |

Table 5: showing significant results from three SNP interaction analysis with dxaxd pattern

|  | SNPi | SNPj | SNPk | unadjust-P | odds_ratio | lower_limit | upper_limit | adjusted-P(fdr) |
| --- | --- | --- | --- | --- | --- | --- | --- | --- |
| 1 | rs1059046 | rs1059047 | rs1130866 | 0.002 | 2.467 | 1.372 | 4.526 | 0.034 |
| 2 | rs1059046 | rs1059057 | rs1130866 | 0.002 | 2.411 | 1.342 | 4.420 | 0.034 |
| 3 | rs1059046 | rs2077079 | rs3024798 | 0.000 | 3.589 | 1.852 | 7.167 | 0.002 |
| 4 | rs1059046 | rs1130866 | rs1124 | 0.002 | 0.437 | 0.246 | 0.765 | 0.034 |
| 5 | rs1059046 | rs4715 | rs721917 | 0.000 | 0.224 | 0.120 | 0.406 | 0.000 |
| 6 | rs1059046 | rs4715 | rs2243639 | 0.001 | 0.428 | 0.244 | 0.739 | 0.031 |
| 7 | rs1059046 | rs1124 | rs721917 | 0.000 | 0.208 | 0.109 | 0.383 | 0.000 |
| 8 | rs1059046 | rs1124 | rs2243639 | 0.000 | 0.322 | 0.180 | 0.563 | 0.001 |
| 9 | rs17886395 | rs1059047 | rs4715 | 0.002 | 0.381 | 0.196 | 0.725 | 0.034 |
| 10 | rs17886395 | rs1059057 | rs4715 | 0.002 | 0.381 | 0.196 | 0.725 | 0.034 |
| 11 | rs17886395 | rs3024798 | rs1130866 | 0.002 | 0.403 | 0.213 | 0.743 | 0.034 |
| 12 | rs1059047 | rs1136450 | rs1130866 | 0.004 | 0.358 | 0.165 | 0.740 | 0.049 |
| 13 | rs1136450 | rs2077079 | rs1124 | 0.001 | 0.378 | 0.203 | 0.688 | 0.025 |
| 14 | rs1136450 | rs7316 | rs4715 | 0.000 | 3.675 | 2.114 | 6.550 | 0.000 |
| 15 | rs1136450 | rs7316 | rs1124 | 0.000 | 4.420 | 2.513 | 7.997 | 0.000 |
| 16 | rs1136450 | rs4715 | rs2243639 | 0.002 | 0.464 | 0.273 | 0.776 | 0.034 |
| 17 | rs1136450 | rs1124 | rs2243639 | 0.002 | 0.459 | 0.268 | 0.774 | 0.034 |
| 18 | rs1136451 | rs1130866 | rs1124 | 0.002 | 0.417 | 0.231 | 0.741 | 0.034 |
| 19 | rs1136451 | rs7316 | rs4715 | 0.002 | 2.172 | 1.295 | 3.686 | 0.034 |
| 20 | rs1136451 | rs4715 | rs721917 | 0.001 | 0.419 | 0.241 | 0.718 | 0.025 |
| 21 | rs1136451 | rs4715 | rs2243639 | 0.001 | 0.411 | 0.238 | 0.699 | 0.021 |
| 22 | rs1136451 | rs1124 | rs721917 | 0.000 | 0.392 | 0.223 | 0.676 | 0.014 |
| 23 | rs1136451 | rs1124 | rs2243639 | 0.001 | 0.414 | 0.240 | 0.703 | 0.021 |
| 24 | rs2077079 | rs3024798 | rs7316 | 0.000 | 5.483 | 2.292 | 14.492 | 0.001 |
| 25 | rs3024798 | rs1130866 | rs4715 | 0.002 | 0.409 | 0.220 | 0.739 | 0.034 |
| 26 | rs3024798 | rs1130866 | rs1124 | 0.003 | 0.421 | 0.227 | 0.763 | 0.036 |
| 27 | rs1130866 | rs4715 | rs1124 | 0.001 | 2.669 | 1.410 | 5.190 | 0.034 |
| 28 | rs1130866 | rs1124 | rs721917 | 0.003 | 2.066 | 1.247 | 3.440 | 0.045 |
| 29 | rs7316 | rs1124 | rs2243639 | 0.000 | 2.838 | 1.581 | 5.207 | 0.009 |

Table 6: showing significant results from three SNP interaction analysis with dxdxd pattern

|  | SNPi | SNPj | SNPk | unadjust-P | odds_ratio | lower_limit | upper_limit | adjusted-P(fdr) |
| --- | --- | --- | --- | --- | --- | --- | --- | --- |
| 1 | rs1059046 | rs17886395 | rs1059047 | 0.002 | 1.719 | 1.214 | 2.449 | 0.007 |
| 2 | rs1059046 | rs17886395 | rs1136450 | 0.001 | 1.756 | 1.256 | 2.468 | 0.003 |
| 3 | rs1059046 | rs17886395 | rs1059057 | 0.005 | 1.634 | 1.153 | 2.331 | 0.018 |
| 4 | rs1059046 | rs17886395 | rs2077079 | 0.000 | 1.807 | 1.312 | 2.499 | 0.001 |
| 5 | rs1059046 | rs17886395 | rs3024798 | 0.005 | 1.565 | 1.133 | 2.170 | 0.019 |
| 6 | rs1059046 | rs17886395 | rs1130866 | 0.010 | 1.502 | 1.091 | 2.074 | 0.033 |
| 7 | rs1059046 | rs17886395 | rs7316 | 0.000 | 0.484 | 0.338 | 0.690 | 0.000 |
| 8 | rs1059046 | rs17886395 | rs1124 | 0.001 | 1.731 | 1.239 | 2.424 | 0.004 |
| 9 | rs1059046 | rs17886395 | rs721917 | 0.009 | 1.528 | 1.101 | 2.133 | 0.030 |
| 10 | rs1059046 | rs1059047 | rs1136450 | 0.000 | 2.045 | 1.460 | 2.884 | 0.000 |
| 11 | rs1059046 | rs1059047 | rs1136451 | 0.001 | 1.713 | 1.227 | 2.404 | 0.005 |
| 12 | rs1059046 | rs1059047 | rs1059057 | 0.008 | 1.598 | 1.119 | 2.296 | 0.028 |
| 13 | rs1059046 | rs1059047 | rs2077079 | 0.012 | 1.503 | 1.090 | 2.078 | 0.037 |
| 14 | rs1059046 | rs1059047 | rs7316 | 0.000 | 0.482 | 0.336 | 0.689 | 0.000 |
| 15 | rs1059046 | rs1059047 | rs721917 | 0.000 | 1.809 | 1.293 | 2.546 | 0.002 |
| 16 | rs1059046 | rs1059047 | rs2243639 | 0.000 | 1.787 | 1.292 | 2.482 | 0.002 |
| 17 | rs1059046 | rs1136450 | rs1136451 | 0.000 | 1.915 | 1.359 | 2.718 | 0.001 |
| 18 | rs1059046 | rs1136450 | rs1059057 | 0.000 | 2.083 | 1.487 | 2.939 | 0.000 |
| 19 | rs1059046 | rs1136450 | rs2077079 | 0.008 | 0.667 | 0.493 | 0.901 | 0.026 |
| 20 | rs1059046 | rs1136450 | rs721917 | 0.000 | 2.052 | 1.453 | 2.921 | 0.000 |
| 21 | rs1059046 | rs1136451 | rs1059057 | 0.003 | 1.636 | 1.171 | 2.298 | 0.012 |
| 22 | rs1059046 | rs1136451 | rs4715 | 0.000 | 0.539 | 0.386 | 0.748 | 0.001 |
| 23 | rs1059046 | rs1059057 | rs2077079 | 0.008 | 1.531 | 1.110 | 2.119 | 0.026 |
| 24 | rs1059046 | rs1059057 | rs3024798 | 0.014 | 1.485 | 1.073 | 2.062 | 0.043 |
| 25 | rs1059046 | rs1059057 | rs7316 | 0.000 | 0.494 | 0.344 | 0.706 | 0.001 |
| 26 | rs1059046 | rs1059057 | rs721917 | 0.000 | 1.842 | 1.317 | 2.594 | 0.002 |
| 27 | rs1059046 | rs1059057 | rs2243639 | 0.000 | 1.823 | 1.318 | 2.533 | 0.001 |
| 28 | rs1059046 | rs2077079 | rs3024798 | 0.016 | 1.458 | 1.070 | 1.993 | 0.046 |
| 29 | rs1059046 | rs2077079 | rs4715 | 0.000 | 3.415 | 2.381 | 4.944 | 0.000 |
| 30 | rs1059046 | rs2077079 | rs1124 | 0.000 | 3.545 | 2.469 | 5.138 | 0.000 |
| 31 | rs1059046 | rs3024798 | rs4715 | 0.000 | 2.293 | 1.637 | 3.229 | 0.000 |
| 32 | rs1059046 | rs3024798 | rs1124 | 0.000 | 2.197 | 1.575 | 3.080 | 0.000 |
| 33 | rs1059046 | rs1130866 | rs4715 | 0.001 | 1.771 | 1.259 | 2.499 | 0.004 |
| 34 | rs1059046 | rs4715 | rs721917 | 0.001 | 0.581 | 0.416 | 0.808 | 0.004 |
| 35 | rs1059046 | rs721917 | rs2243639 | 0.005 | 1.549 | 1.133 | 2.127 | 0.019 |
| 36 | rs17886395 | rs1059047 | rs1136450 | 0.001 | 1.775 | 1.250 | 2.536 | 0.004 |
| 37 | rs17886395 | rs1059047 | rs1136451 | 0.000 | 1.995 | 1.365 | 2.946 | 0.002 |
| 38 | rs17886395 | rs1059047 | rs1059057 | 0.001 | 1.934 | 1.293 | 2.925 | 0.004 |
| 39 | rs17886395 | rs1059047 | rs1130866 | 0.002 | 1.673 | 1.203 | 2.337 | 0.007 |
| 40 | rs17886395 | rs1059047 | rs721917 | 0.015 | 1.537 | 1.081 | 2.199 | 0.044 |
| 41 | rs17886395 | rs1059047 | rs2243639 | 0.006 | 1.569 | 1.124 | 2.201 | 0.023 |
| 42 | rs17886395 | rs1136450 | rs1136451 | 0.000 | 2.182 | 1.546 | 3.104 | 0.000 |
| 43 | rs17886395 | rs1136450 | rs1059057 | 0.003 | 1.685 | 1.186 | 2.410 | 0.011 |
| 44 | rs17886395 | rs1136450 | rs1130866 | 0.001 | 1.717 | 1.250 | 2.367 | 0.003 |
| 45 | rs17886395 | rs1136451 | rs1059057 | 0.000 | 2.148 | 1.471 | 3.169 | 0.000 |
| 46 | rs17886395 | rs1136451 | rs1130866 | 0.000 | 2.941 | 2.070 | 4.217 | 0.000 |
| 47 | rs17886395 | rs1136451 | rs2243639 | 0.004 | 1.588 | 1.150 | 2.201 | 0.016 |
| 48 | rs17886395 | rs1059057 | rs1130866 | 0.000 | 1.827 | 1.310 | 2.562 | 0.002 |
| 49 | rs17886395 | rs1059057 | rs2243639 | 0.015 | 1.499 | 1.073 | 2.104 | 0.045 |
| 50 | rs17886395 | rs2077079 | rs3024798 | 0.000 | 2.006 | 1.460 | 2.767 | 0.000 |
| 51 | rs17886395 | rs2077079 | rs7316 | 0.000 | 2.012 | 1.419 | 2.864 | 0.000 |
| 52 | rs17886395 | rs2077079 | rs4715 | 0.000 | 2.377 | 1.686 | 3.367 | 0.000 |
| 53 | rs17886395 | rs2077079 | rs1124 | 0.000 | 2.946 | 2.084 | 4.191 | 0.000 |
| 54 | rs17886395 | rs3024798 | rs1130866 | 0.000 | 1.778 | 1.284 | 2.468 | 0.002 |
| 55 | rs17886395 | rs3024798 | rs4715 | 0.000 | 1.879 | 1.346 | 2.632 | 0.001 |
| 56 | rs17886395 | rs3024798 | rs1124 | 0.000 | 2.183 | 1.564 | 3.059 | 0.000 |
| 57 | rs17886395 | rs7316 | rs2243639 | 0.004 | 1.639 | 1.161 | 2.318 | 0.016 |
| 58 | rs17886395 | rs4715 | rs1124 | 0.001 | 1.775 | 1.263 | 2.504 | 0.003 |
| 59 | rs17886395 | rs1124 | rs2243639 | 0.007 | 1.560 | 1.118 | 2.183 | 0.025 |
| 60 | rs17886395 | rs721917 | rs2243639 | 0.000 | 1.864 | 1.337 | 2.616 | 0.001 |
| 61 | rs1059047 | rs1136450 | rs1136451 | 0.000 | 2.093 | 1.481 | 2.980 | 0.000 |
| 62 | rs1059047 | rs1136450 | rs1059057 | 0.000 | 1.920 | 1.336 | 2.781 | 0.002 |
| 63 | rs1059047 | rs1136450 | rs721917 | 0.012 | 1.506 | 1.088 | 2.095 | 0.037 |
| 64 | rs1059047 | rs1136451 | rs1059057 | 0.001 | 1.839 | 1.252 | 2.726 | 0.005 |
| 65 | rs1059047 | rs1136451 | rs1130866 | 0.000 | 1.853 | 1.317 | 2.623 | 0.002 |
| 66 | rs1059047 | rs1136451 | rs721917 | 0.000 | 2.004 | 1.406 | 2.881 | 0.000 |
| 67 | rs1059047 | rs1136451 | rs2243639 | 0.000 | 2.015 | 1.436 | 2.847 | 0.000 |
| 68 | rs1059047 | rs1059057 | rs1130866 | 0.000 | 2.303 | 1.621 | 3.296 | 0.000 |
| 69 | rs1059047 | rs2077079 | rs3024798 | 0.000 | 2.360 | 1.707 | 3.277 | 0.000 |
| 70 | rs1059047 | rs2077079 | rs7316 | 0.001 | 1.826 | 1.278 | 2.619 | 0.003 |
| 71 | rs1059047 | rs2077079 | rs4715 | 0.001 | 1.729 | 1.226 | 2.446 | 0.005 |
| 72 | rs1059047 | rs2077079 | rs1124 | 0.000 | 2.175 | 1.540 | 3.085 | 0.000 |
| 73 | rs1059047 | rs3024798 | rs1124 | 0.009 | 1.544 | 1.104 | 2.164 | 0.029 |
| 74 | rs1059047 | rs3024798 | rs721917 | 0.007 | 1.552 | 1.118 | 2.164 | 0.024 |
| 75 | rs1059047 | rs1130866 | rs2243639 | 0.013 | 1.510 | 1.087 | 2.104 | 0.040 |
| 76 | rs1059047 | rs4715 | rs1124 | 0.000 | 2.177 | 1.543 | 3.086 | 0.000 |
| 77 | rs1059047 | rs721917 | rs2243639 | 0.000 | 2.238 | 1.600 | 3.154 | 0.000 |
| 78 | rs1136450 | rs1136451 | rs1059057 | 0.000 | 1.996 | 1.411 | 2.842 | 0.000 |
| 79 | rs1136450 | rs1136451 | rs4715 | 0.005 | 0.638 | 0.461 | 0.881 | 0.018 |
| 80 | rs1136450 | rs1059057 | rs721917 | 0.008 | 1.533 | 1.107 | 2.133 | 0.026 |
| 81 | rs1136450 | rs2077079 | rs3024798 | 0.000 | 2.592 | 1.870 | 3.617 | 0.000 |
| 82 | rs1136450 | rs2077079 | rs1130866 | 0.000 | 2.117 | 1.538 | 2.925 | 0.000 |
| 83 | rs1136450 | rs3024798 | rs1130866 | 0.000 | 2.096 | 1.527 | 2.885 | 0.000 |
| 84 | rs1136450 | rs3024798 | rs721917 | 0.016 | 0.690 | 0.508 | 0.937 | 0.046 |
| 85 | rs1136450 | rs7316 | rs4715 | 0.010 | 1.572 | 1.105 | 2.245 | 0.033 |
| 86 | rs1136450 | rs7316 | rs1124 | 0.000 | 2.569 | 1.782 | 3.730 | 0.000 |
| 87 | rs1136450 | rs4715 | rs1124 | 0.005 | 1.615 | 1.146 | 2.288 | 0.019 |
| 88 | rs1136450 | rs721917 | rs2243639 | 0.000 | 2.212 | 1.599 | 3.079 | 0.000 |
| 89 | rs1136451 | rs1059057 | rs1130866 | 0.000 | 2.018 | 1.431 | 2.864 | 0.000 |
| 90 | rs1136451 | rs1059057 | rs721917 | 0.000 | 1.915 | 1.342 | 2.755 | 0.001 |
| 91 | rs1136451 | rs1059057 | rs2243639 | 0.000 | 1.930 | 1.374 | 2.727 | 0.001 |
| 92 | rs1136451 | rs2077079 | rs3024798 | 0.001 | 1.661 | 1.219 | 2.271 | 0.004 |
| 93 | rs1136451 | rs2077079 | rs1130866 | 0.001 | 1.733 | 1.243 | 2.423 | 0.004 |
| 94 | rs1136451 | rs2077079 | rs4715 | 0.001 | 1.731 | 1.238 | 2.428 | 0.004 |
| 95 | rs1136451 | rs2077079 | rs1124 | 0.000 | 2.109 | 1.508 | 2.961 | 0.000 |
| 96 | rs1136451 | rs3024798 | rs1130866 | 0.000 | 1.858 | 1.342 | 2.581 | 0.001 |
| 97 | rs1136451 | rs3024798 | rs4715 | 0.005 | 1.595 | 1.146 | 2.227 | 0.018 |
| 98 | rs1136451 | rs3024798 | rs1124 | 0.000 | 1.847 | 1.328 | 2.577 | 0.001 |
| 99 | rs1136451 | rs1130866 | rs721917 | 0.000 | 1.773 | 1.280 | 2.469 | 0.003 |
| 100 | rs1136451 | rs1130866 | rs2243639 | 0.006 | 1.556 | 1.128 | 2.151 | 0.023 |
| 101 | rs1136451 | rs7316 | rs1124 | 0.004 | 1.659 | 1.166 | 2.369 | 0.015 |
| 102 | rs1136451 | rs7316 | rs2243639 | 0.006 | 1.584 | 1.128 | 2.227 | 0.021 |
| 103 | rs1136451 | rs4715 | rs1124 | 0.009 | 1.548 | 1.106 | 2.175 | 0.030 |
| 104 | rs1059057 | rs2077079 | rs3024798 | 0.000 | 2.409 | 1.741 | 3.348 | 0.000 |
| 105 | rs1059057 | rs2077079 | rs7316 | 0.000 | 1.875 | 1.310 | 2.693 | 0.002 |
| 106 | rs1059057 | rs2077079 | rs4715 | 0.001 | 1.729 | 1.226 | 2.446 | 0.005 |
| 107 | rs1059057 | rs2077079 | rs1124 | 0.000 | 2.224 | 1.574 | 3.158 | 0.000 |
| 108 | rs1059057 | rs3024798 | rs7316 | 0.012 | 1.562 | 1.089 | 2.248 | 0.036 |
| 109 | rs1059057 | rs3024798 | rs1124 | 0.007 | 1.577 | 1.127 | 2.212 | 0.024 |
| 110 | rs1059057 | rs3024798 | rs721917 | 0.005 | 1.582 | 1.139 | 2.206 | 0.020 |
| 111 | rs1059057 | rs4715 | rs1124 | 0.000 | 2.177 | 1.543 | 3.086 | 0.000 |
| 112 | rs1059057 | rs721917 | rs2243639 | 0.000 | 2.276 | 1.626 | 3.207 | 0.000 |
| 113 | rs2077079 | rs3024798 | rs4715 | 0.001 | 1.714 | 1.244 | 2.369 | 0.004 |
| 114 | rs2077079 | rs3024798 | rs1124 | 0.001 | 1.651 | 1.198 | 2.282 | 0.007 |
| 115 | rs2077079 | rs7316 | rs721917 | 0.001 | 1.809 | 1.278 | 2.572 | 0.003 |
| 116 | rs3024798 | rs4715 | rs1124 | 0.000 | 2.549 | 1.787 | 3.668 | 0.000 |
| 117 | rs3024798 | rs4715 | rs721917 | 0.002 | 1.651 | 1.188 | 2.302 | 0.010 |
| 118 | rs3024798 | rs1124 | rs721917 | 0.011 | 1.505 | 1.088 | 2.088 | 0.035 |
| 119 | rs1130866 | rs4715 | rs2243639 | 0.013 | 1.521 | 1.086 | 2.135 | 0.041 |
| 120 | rs1130866 | rs721917 | rs2243639 | 0.001 | 1.731 | 1.248 | 2.414 | 0.004 |
| 121 | rs7316 | rs4715 | rs1124 | 0.002 | 0.577 | 0.406 | 0.819 | 0.007 |
| 122 | rs7316 | rs4715 | rs721917 | 0.003 | 1.686 | 1.180 | 2.421 | 0.012 |
| 123 | rs7316 | rs4715 | rs2243639 | 0.000 | 2.447 | 1.694 | 3.559 | 0.000 |
| 124 | rs7316 | rs1124 | rs721917 | 0.002 | 1.738 | 1.215 | 2.501 | 0.007 |
| 125 | rs7316 | rs1124 | rs2243639 | 0.000 | 2.693 | 1.861 | 3.927 | 0.000 |
| 126 | rs1124 | rs721917 | rs2243639 | 0.010 | 1.500 | 1.092 | 2.064 | 0.033 |
